# Coevolution between Male and Female Genitalia in the *Drosophila melanogaster* Species Subgroup

**DOI:** 10.1371/journal.pone.0057158

**Published:** 2013-02-25

**Authors:** Amir Yassin, Virginie Orgogozo

**Affiliations:** Centre National de la Recherche Scientifique - Unité Mixte de Recherche 7592, Institut Jacques Monod, Université Paris Diderot, Paris, France; Australian Museum, Australia

## Abstract

In contrast to male genitalia that typically exhibit patterns of rapid and divergent evolution among internally fertilizing animals, female genitalia have been less well studied and are generally thought to evolve slowly among closely-related species. As a result, few cases of male-female genital coevolution have been documented. In *Drosophila*, female copulatory structures have been claimed to be mostly invariant compared to male structures. Here, we re-examined male and female genitalia in the nine species of the *D. melanogaster* subgroup. We describe several new species-specific female genital structures that appear to coevolve with male genital structures, and provide evidence that the coevolving structures contact each other during copulation. Several female structures might be defensive shields against apparently harmful male structures, such as cercal teeth, phallic hooks and spines. Evidence for male-female morphological coevolution in *Drosophila* has previously been shown at the post-copulatory level (e.g., sperm length and sperm storage organ size), and our results provide support for male-female coevolution at the copulatory level.

## Introduction

In most animal species with internal fertilization, male external genitalia are the most rapidly evolving organs and they usually are the first organs to diverge morphologically following speciation [1]. Because of their rapid evolution and species-specificity, their illustration is a common feature of taxonomic literature to discriminate closely-related species. Among the various hypotheses proposed to explain such a rapid male genitalia evolution, two appear as the most plausible [2]. First, the cryptic female choice (CFC) hypothesis postulates that male genitalia evolution is driven by the ‘aesthetic’ sense of females [1]. This hypothesis considers the great diversity of male external genitalia comparable to the rapid evolution of exaggerated sexual ornaments (e.g. feather colors) that are used to charm or lure females. Second, the sexually antagonistic coevolution (SAC) hypothesis postulates that the reproductive optimum of one sex is in opposition to that of the other, setting up an escalating arms race of antagonistic traits in males and females. Morphological traits under SAC include male genitalia that cause damage to the female, in order to directly or indirectly maximize the use of the male’s own sperm, in particular by preventing females from remating [3]–[5].

The coevolution between male and female genitalia expected under CFC differs from the one expected under SAC [2]. On one hand, CFC predicts that female changes will probably involve physiological and neuronal aspects. These postulated yet unknown female modifications should be unraveled by future neurobiological research, such as examinations of female reproductive tract neurons. CFC is also compatible with a certain degree of morphological coevolution between male and female genitalia, which would be on a “cooperative basis”, such as grooves and furrows helping males to grasp the female, or helping females to sense the male. Such a pattern of cooperative coevolution has been widely documented in Pholcidae spiders between male cheliceral apophyses and female epigynal pockets [6]–[8]. On the other hand, SAC predicts that female genitalia might evolve in response to male aggressive genital structures on a “defensive basis” in order to resist the harm induced by males. Few instances of resistant female structures coevolving with male harmful genitalia have been documented and even fewer appear to be defensive [2], [9]. These examples include the genital pads in Malabar ricefish [10], [11], the thickness of vaginal connective tissues in seed beetles [12], the genital spines in water striders [13], the paragenital systems in bedbugs [14], the vaginal coils in waterfowl [15] and morphometrical covariations in female guppies [16] and dung beetles [17]. Most of these cases involve species with coercive mating and reduced courtship, suggesting that the lack of female ‘aesthetic’ senses in these species may have led to the evolution of such cases [18]–[20].

Two comparative studies of genitalia in various fruit flies of the genus *Drosophila* concluded that in contrast to rapidly evolving male genitalia, female genital morphology is “practically invariable” among closely-related species that have diverged 3 million years ago (Ma) [21], and that their general form remained identical between distantly-related species that have diverged 40–60 Ma [22]. Because courtship is elaborate in *Drosophila* species and involves different aspects that appear to influence female choice [23], CFC has been thought to be the primary factor explaining the rapid evolution of male genitalia in these flies [21]. However, *Drosophila* copulation anatomy has recently been investigated in detail, and a general pattern seems to emerge, with male genitalia causing copulatory wounds to the female tract, mainly via phallic auxiliary organs known as posterior parameres or inner paraphyses [24], [25] or via phallic spikes [26]. Whether these wounds reduce survival of mated females is unknown, although they were shown to trigger a localized immune response [27]. In *D. melanogaster*, a few harmful seminal proteins such as the sex peptide are known to enter the female hemolymph through the intima of the anterior margin of the vagina [28], [29] where the mating wounds form [25]. Comparative investigations of copulation anatomy between species also revealed two female genital structures coevolving with male parts. First, in the four species of the *melanogaster* complex, a membraneous pleural pouch before the anterior margin of the female oviscapt (sternite 8) and below tergite 8 harbors the male epandrial posterior lobes at the late stages of copulation [30], [31]. The size of this female pouch covaries with male lobe size between the four species. Second, in two species of the *yakuba* complex, a furrow at the antero-dorsal margin of the oviscapt harbors the male phallic basal spikes during intromission [26], [27]. The sizes of these female furrows and male spikes also covary between species of the *yakuba* complex.

We conducted here a detailed comparative analysis of male and female genitalia in the nine species of the *melanogaster* subgroup. We found several new female characters whose evolution between species correlates with changes in contacting male structures.

## Materials and Methods

### Fly Culture and Morphological Analyses

Males and females were obtained from laboratory cultures of the nine species of the *melanogaster* subgroup (Table 1) and reared on standard *Drosophila* medium at 21°C. Cultures were kindly provided by Jean R. David (CNRS, Gif-sur-Yvette) and we confirmed the identification of each species based on species-specific male genitalia traits [32]–[38]. Genitalia of at least 10 individuals per sex and per species were dissected, mounted on microscopic slides in DMHF mounting medium (Entomopraxis A9001) and photographed under a Keyence VHX-2000 light microscope. Outlines of male epandrial posterior lobes and female oviscapt pouches were drawn manually on the light microscope images and their areas were estimated with the ImageJ software package [39]. Measurements were taken on well-dissected and correctly oriented preparations for a single pouch per female (*D. melanogaster*, *N* = 17; *D. simulans*, *N* = 20; and *D. sechellia*, *N* = 19) and from a single epandrial posterior lobe per male (*D. melanogaster*, *N* = 8; *D. simulans*, *N* = 9; and *D. sechellia*, *N* = 5). In addition, 10 *D. simulans* virgin females were examined for the presence of an oviscapt pouch. These virgin females were selected at the pupal stage based on sex comb absence and adults were grown on standard food for 8 days before dissection. Scanning electron microscopy (SEM) was performed using standard protocol.

**Table 1:** Geographical origin and date of collection of the nine laboratory strains used in this study.

Species	Geographical origin	Collection date	Collector	*Drosophila* San DiegoStock Center number
*D. melanogaster*	Marrakech, Morocco	2009	Jean R. David	
*D. simulans*	Marrakech, Morocco	2009	Jean R. David	
*D. sechellia*	Seychelles Islands	1985	Unknown	
*D. mauritiana*	Mauritius Island	1985	Unknown	
*D. teissieri*	Mt Selinda, Zimbabwe	1970H. E. Paterson		
*D. yakuba*	Andasibe, Madagascar	2008	Jean R. David & Amir Yassin	
*D. santomea*	São Tomé Island	1998	Daniel Lachaise	14021-0271.00
*D. erecta*	Lamto, Côte d’Ivoire	1971	Daniel Lachaise	
*D. orena*	Bafut N’Guemba,Cameroon	1975	Jean R. David, Daniel Lachaise& Léonidas Tsacas	14021-0245.01

For the two species of the *melanogaster* subgroup whose copulation anatomy has never been described, *D. orena* and *D. erecta*, pairs were dissected *in copula* to investigate the position of male and female genital structures during mating. For each species, 20 virgin females were kept in a vial for five days, and then mated *en masse* to 4–5 days old males. Ten tubes (*N* = 200 females) were used for each species. At 3–5 minutes from the start of matings, flies were killed by ether and conserved in absolute ethanol. Thirty mating pairs were dissected, mounted in DMHF and observed under a Leica DMZ light microscope for each species. Ether has also been used efficiently to kill copulating pairs in several other species of the *D. melanogaster* subgroup (Jean David, personal communication) and *D. orena* flies were killed as rapidly as *D. erecta* in presence of ether. We therefore think that the superficial penetration in *D. orena* is not an artifact due to rapid withdrawal of their genitalia before death.

### Phylogenetic Analysis of Male-female Genital Coevolution

Coevolution between male and female structures was inferred using Pagel’s [40] phylogenetic correlation (λ) test as implemented in the MESQUITE software package [41]. Male and female characters were binary coded (0 = absent, 1 = present) and mapped on the phylogenetic tree of the nine species inferred from Obbard et al. [42] (File S1). For each characters pair, likelihood ratios are compared between two models, one with independent rates of character evolution and the other with the rate of one character depending on that of the second character. Significance was estimated from simulation data after 100 or 1000 iterations using MESQUITE, and False Discovery Rate (FDR) control [43] was applied to correct for multiple comparisons, as implemented in the LBE 1.22 software package in R [44].

## Results

### Species-specific Female Genitalia

In contrast to previous reports [21], [22], our detailed examination of the nine species of the *D. melanogaster* subgroup uncovered several novel female genitalia structures that are species-specific. These female structures can be classified under two categories: external pouches and internal vaginal shields. We discovered sclerotized depressions of distinctive sizes and shapes at the postero-dorsal margin of the oviscapt in five species. They differ from the membraneous pleural pouches described previously by Robertson [30] and Kamimura and Mitsumuto [31] that are located anteriorly at the junction between the oviscapt and the eighth tergite. These newly described sclerotized structures were recently found independently by Kamimura and Mitsumuto [26] in two species, *D. yakuba* and *D. teissieri*. Furthermore, we detected sclerifications on internal walls of the vagina, that we named vaginal shields, in three species. Those of *D. orena* were previously described by Tsacas and David [35]. We provide below a detailed account of these female structures.

To identify the male parts that contact these female structures during copulation, we examined the anatomy of copulating pairs. Based on previous reports for seven *D. melanogaster* subgroup species [21], [22], [25], [26], [30], [31] and our observations for two species for which no data were available, we identified male organs that contact each female structure during copulation. Phylogenetic correlation analysis revealed significant correlated evolution of these interacting male and female genitalia structures in the *D. melanogaster* subgroup.

### Female Oviscapt Pouches

**Figure 1:**
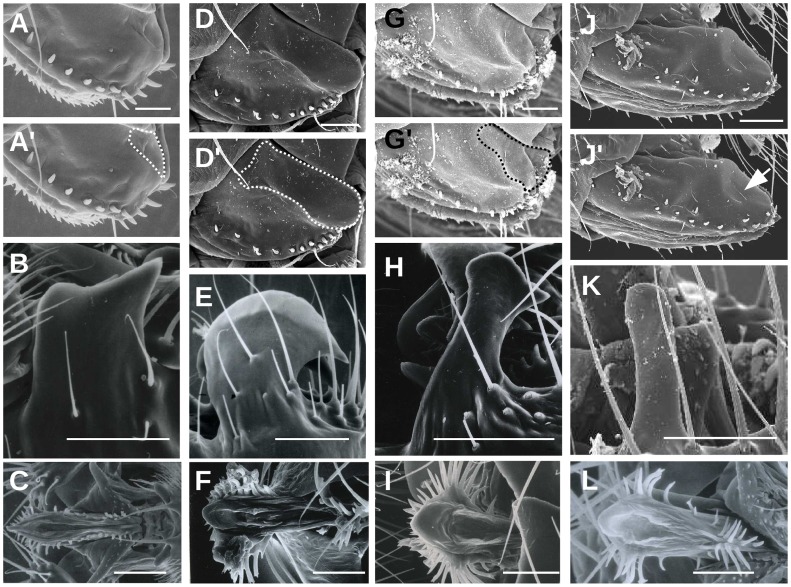
Scanning electron micrographs of female oviscapts (A, D, G, J) and male epandrial posterior lobes (B, E, H, K) and phalli (C, F, I, L) in species of the *melanogaster* complex: *D. melanogaster* (A, C), *D. simulans* (D–F), *D. sechellia* (G–I) and *D. mauritiana* (J–L). Each oviscapt picture is duplicated, with the oviscapt pouch contours outlined in (A′, D′, G′, J′). Note the presence of a slight depression on the oviscapt of *D. mauritiana* (J′; arrow), suggesting that a small pouch may exist in this species (see text). Scale bar is 50 µm.

In a monograph on European drosophilids, Bächli et al. [38] noted the presence of a large depression at the postero-dorsal margin of the oviscapt of *D. simulans* that they suggested to “hold the large male epandrial posterior lobe during copulation.” We examined the oviscapt of *D. simulans* and observed a large depression as indicated by Bächli et al. [38], named hereafter oviscapt pouch (Fig. 1D–D′). This pouch was present in both virgin (*N* = 10) and mated females (*N* = 10). We also examined the remaining three species of the *melanogaster* complex and found smaller oviscapt pouches in two species, *D. melanogaster* (Fig. 1A–A′) and *D. sechellia* (Fig. 1G–G′) and no pouch in *D. mauritiana* (Fig. 1J–J′; *N* = 10).

**Figure 2:**
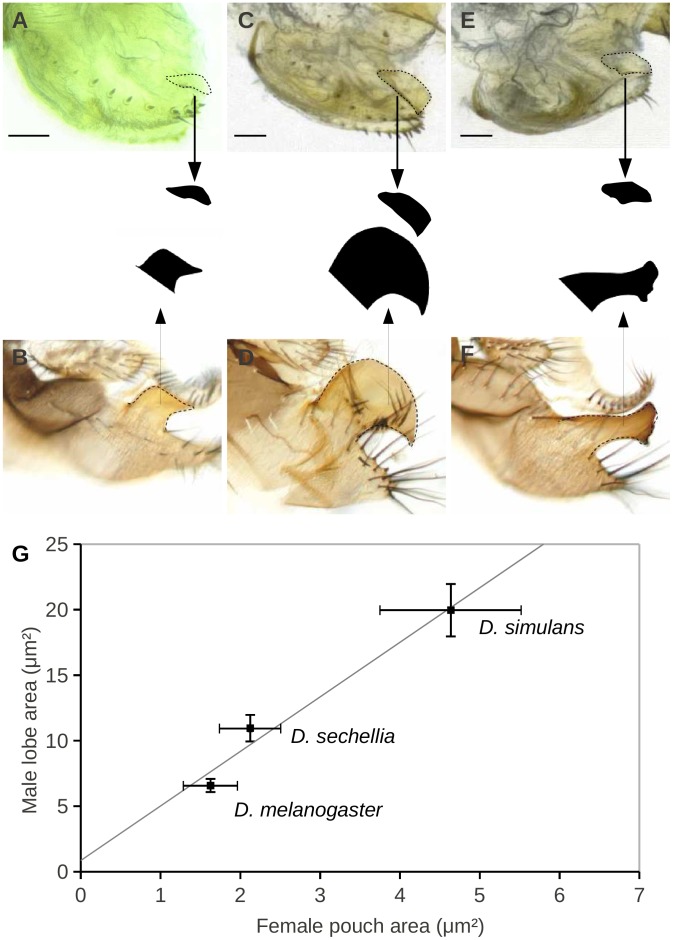
Photomicrographs of female oviscapts (A, C, E) and male epandria (B, D, F) in three species of the *melanogaster* complex: *D. melanogaster* (A, B), *D. simulans* (C, D) and *D. sechellia* (E, F). Oviscapt pouches and epandrial posterior lobes were outlined and the area of their black duplicates was measured. Areas of both structures are significantly correlated between the three species (G). Each point indicates the species average and bars indicate standard deviation. Scale bar is 50 µm.

Mating descriptions in species of the *melanogaster* complex [21], [22], [25], [30], [31] indicate that at the beginning of copulation the postero-dorsal margin of the oviscapt contacts male grasping organs known as epandrial posterior lobes. Epandrial posterior lobes provide the strongest discriminatory characters between species of the *melanogaster* complex (Fig. 1B–C, E–F, H–I, K–L) and have been subject to extensive investigations aiming at identifying the genetic basis of morphological divergence [45]–[50]. We found that average female pouch area correlates with average male lobe area in the *melanogaster* complex species (Spearman’s rank correlation: *r* = 1.00, *P*<0.157; Fig. 2). In *D. mauritiana,* the epandrial posterior lobe is reduced to a small rod (Fig. 1K). Although a slight depression at the postero-dorsal margin of *D. mauritiana* oviscapt might be perceptible on SEM photos (arrow in Fig. 1J′), we did not detect any oviscapt pouch in dissected *D. mauritiana* oviscapts under a conventional light microscope.

### Female Oviscapt Furrows

**Figure 3:**
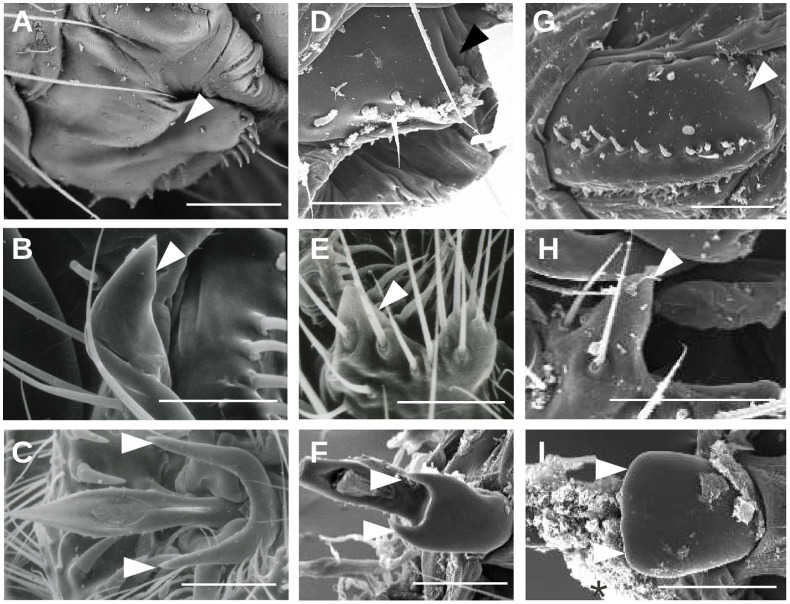
Micrographs of female oviscapts (A, D, G) and male epandrial posterior lobes (B, E, H) and phalli (C, F, I ) in species of the *yakuba* complex: *D. teissieri* (A–C), *D. yakuba* (D–F) and *D. santomea* (G–I); Oviscapt furrows and phallic spurs are indicated by arrowheads. Note the absence of species-specific structures in the male and female genitalia of *D. santomea* (G, I). Scale bar is 50 µm.

In the *yakuba* complex, we also detected a depression at the postero-dorsal margin of the oviscapt in *D. teissieri* (white arrowheads in Fig. 3A) and in *D. yakuba* (Fig. 3D) but not in *D. santomea* (Fig. 3G; *N* = 10). Similar observations were made independently by Kamimura and Mitsumuto [26] in these three species. This depression forms a slit in *D. teissieri* (Fig. 3A) and an oval pocket in *D. yakuba* (Fig. 3D, see also Fig. 1e–e′ in Kamimura and Mitsumuto [26]) and is called hereafter oviscapt furrow, as it lacks the oval shape typical of the oviscapt pouches of the *melanogaster* complex.

Small protrusions were also detected in *D. teissieri*, *D. yakuba* and *D. santomea* males in the part of the epandrium that harbors epandrial posterior lobes in species of the *melanogaster* complex (Fig. 3B, E, H). These structures can thus be considered as small epandrial lobes. Lobes of *D. teissieri* (Fig. 3B; [32]) are larger than those of *D. yakuba* (Fig. 3E; [51]), while those of *D. santomea* (Fig. 3H, not reported previously) are of equal size to those of *D. yakuba*. Kamimura and Mitsumuto [26] did not describe the role of these lobes during copulation, but according to their microscopic preparations of mating couples, these lobes do not contact female oviscapt furrows during copulation. The female oviscapt furrows of *D. yakuba* were shown to hold two basal phallic processes during copulation that Kamimura and Mitsumuto [26] called phallic spikes. Phallic spikes are longer in *D. teissieri* than in *D. yakuba* and are absent in *D. santomea* (Fig. 3C, F, I, [26]). The elongated slit-like shape of the *D. teissieri* furrows suggests that, like in *D. yakuba*, they hold phallic spikes during copulation. In the four species of the *melanogaster* complex, no phallic spikes are found and the female pouches contact male epandrial posterior lobes during copulation [21], [25], [31].

**Figure 4:**
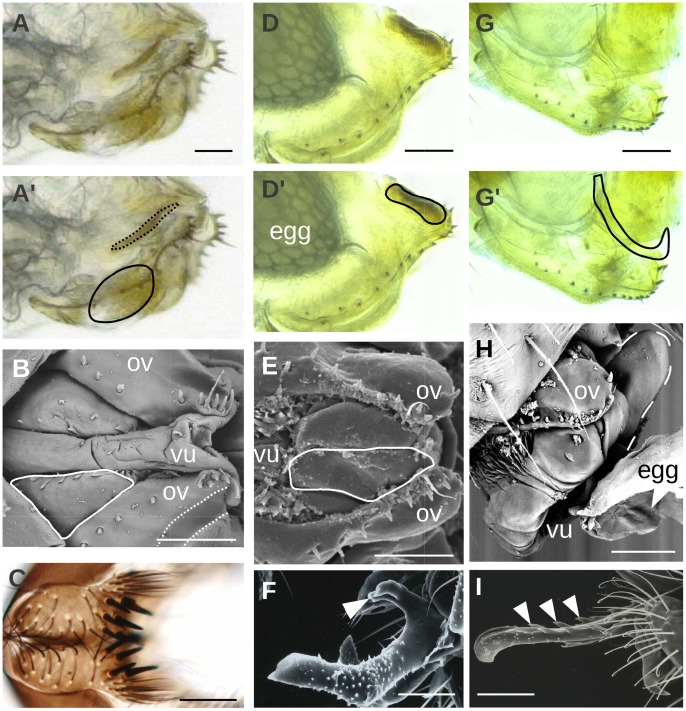
Micrographs of female vaginal shields (A–B, D–E, G–H) and male cerci (C) and phalli (F, I) in *D. teissieri* (A–C), *D. orena* (D–F) and *D. erecta* (G–I). White arrowheads indicate apparently harmful male phallic structures. Each oviscapt picture is duplicated (A′, D′, G′), with the contours of the vaginal shields and oviscapt pouches outlined with continuous and dotted lines, respectively; ov: oviscapt; vu: vulva. Scale bar is 50 µm.

In *D. orena* and *D. erecta,* no female oviscapt depressions were found (*D. orena, N* = 10, Fig. 4D–D′, [35]; *D. erecta*, *N* = 10, Fig. 4G–G′, [33]), nor male epandrial posterior lobes (data not shown). The phalli of these species are the largest among the *melanogaster* subgroup species [52]. Phalli of the *erecta* complex strongly discriminate the two species, and their basal protrusions are different from each others and from the phallic spurs of the *yakuba* complex (Fig. 4F, I). We called these protrusions phallic hooks in *D. orena* (Fig. 4F) and phallic spines in *D. erecta* (Fig. 4I).

### Female Vaginal Shields

Our microscopic investigation of the internal morphology of female genitalia revealed strong sclerites (hereafter vaginal shields) that are found only in *D. teissieri*, *D. erecta* and *D. orena*. In *D. teissieri*, these sclerites are located at the ventral margin of the vagina (Fig. 4A–A′, B); hereafter ventral vaginal shields) and absent from the vagina of its two closely-related species *D. yakuba* and *D. santomea*. During copulation, this part of the vagina contacts male cerci in the four species of the *melanogaster* complex (Fig. 6 in Eberhard and Ramirez [22]; [18], [22], [26]). Interestingly, *D. teissieri* male cerci harbor a set of teeth that are stronger and stouter than in the other species of the *D. melanogaster* subgroup (Fig. 4C; [32]), and whose number and disposition differ among geographically isolated populations [53], [54]. Vaginal shields in this species may thus have evolved as a protection against those strong cercal teeth.

In *D. orena*, we found a sclerification above the female vulva (Fig. 4D–D′, E; hereafter vulval shield; [35]). In *D. erecta*, we found a large sclerite at the dorsal margin of the vaginal duct leading to the uterus (Fig. 4G–G′, H; hereafter uterine shield).

### Copulation Anatomy of *D. orena* and *D. erecta*

**Figure 5:**
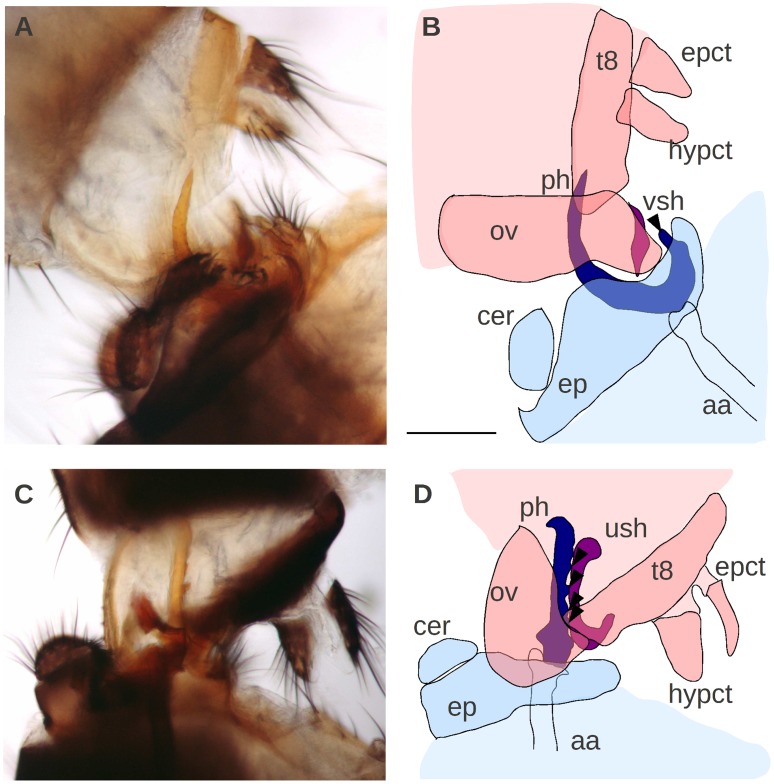
Copulation anatomy of *D. orena* (A–B) and *D. erecta* (C–D). Male and female organs are depicted in blue and pink, respectively, with contacting species-specific structures in dark colors. Note that phallic hooks and spines (arrowheads) contact female vaginal shields during copulation; aa: aedeagal apodeme; cer: cercus; ep: epandrium; epct: epiproct; hypct: hypoproct; ov: oviscapt; ph: phallus; t8: tergite 8; ush: uterine shield; vsh: vulval shield. Note that the male surstyli that grasp the female oviscapt at the ventro-distal margin and the phallic paraphyses were not reproduced in the schematic drawings (B, D) for the sake of clarity. Scale bar is 1 mm.

To determine which male parts come into contact with the vaginal shields in *D. orena* and *D. erecta*, we mounted copulating pairs at 3–5 minutes after copulation started and examined their anatomy. General patterns of the copulation anatomy of *D. orena* and *D. erecta* resembles those of the remaining species of the subgroup (Fig. 5). As in the other species of the subgroup [21], [22], [25], [26], [30], [31], the male abdomen bends at 180° to penetrate the female and the epandrial lobes, which lack epandrial posterior lobes, grasp female oviscapts at the dorso-distal margins while the surstyli grasp them on the ventro-distal margins. The male cerci grasp the female oviscapt at their ventro-medial margin. The male phallus and the two pairs of paraphyses (the inner and outer pairs) penetrate the female vagina. Like in other species [25], [26], [31], the paraphyses spread into the female vagina laterally, with the outer pairs pressing on the female dorso-lateral walls and the inner pairs pressing on her ventro-lateral walls. Phallic penetration was deep in *D. erecta* (Fig. 5C) and superficial in *D. orena* (Fig. 5A). Accordingly, most copulating pairs of *D. orena* fixed in alcohol separated from each other during dissection (17 out of 30 pairs), in contrast to *D. erecta* pairs which were strongly fixed and never detached from each other (*N* = 30 pairs). Our observations show that species-specific vaginal shields in *D. orena* and *D. erecta* contact species-specific phallic hooks and spines, respectively, during copulation (arrowheads in Fig. 5B, D).

**Figure 6:**
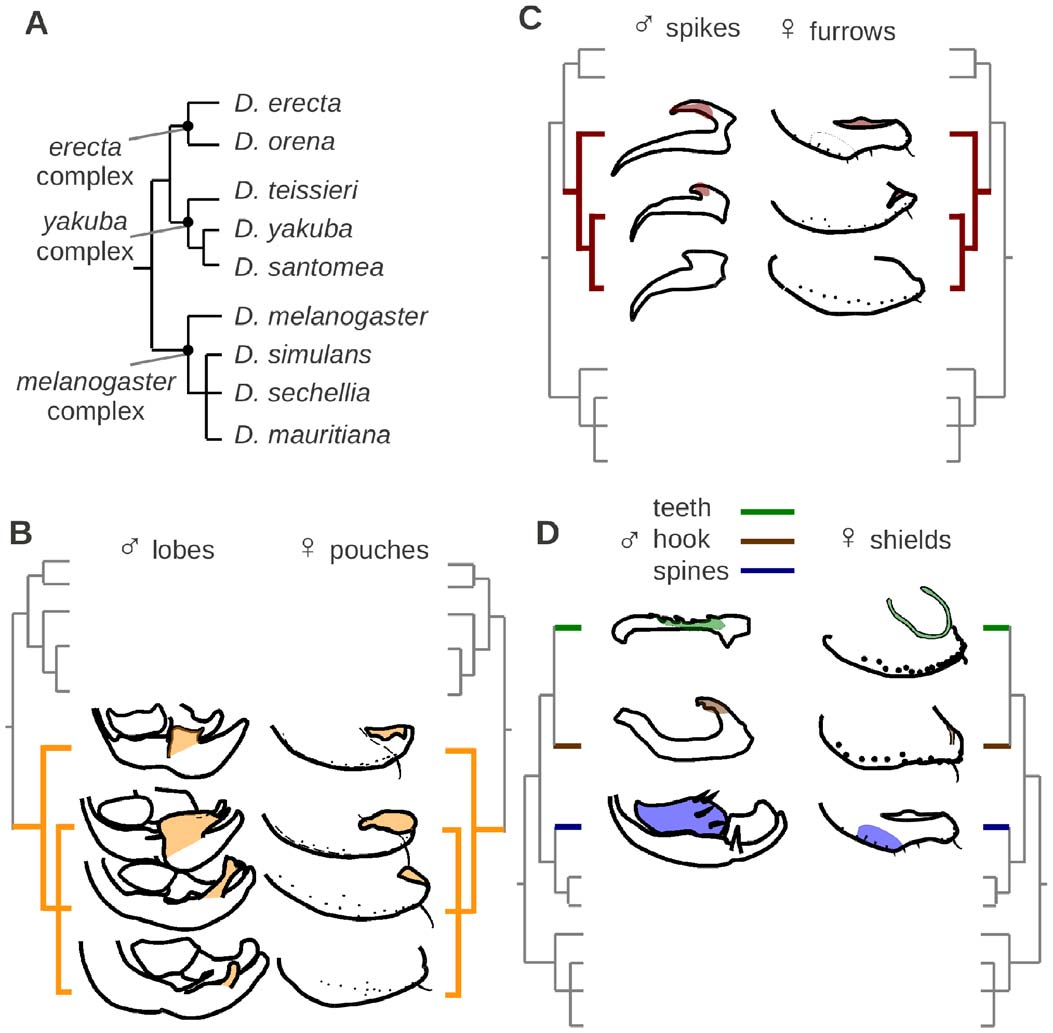
Mapping of male-female genital coevolution on the phylogeny of the nine species of the *melanogaster* subgroup (A) drawn after Obbard et al. [42]: male epandrial posterior lobes and female oviscapt pouches in the melanogaster species complex (B), male phallic spikes and female oviscapt furrows in the yakuba species complex (C), and male phallic spines and hooks and cercal teeth and female uterine, vulval and vaginal shields in *D. erecta*, *D. orena* and *D. teissieri*, respectively (D).

### Phylogenetic Analysis of Coevolution

Male and female genital traits (presence/absence) were mapped on the phylogeny of the nine species in order to test their coevolution (File S1; Fig. 6). Table 2 shows the distribution of Pagel’s phylogenetic correlations (λ) between the different male and female genital structures described here, and their corresponding probability values after FDR correction for multiple comparisons. With the exception of the negative correlation between male epandrial posterior lobes in the *melanogaster* complex and the small lobes of the *yakuba* complex (λ = 3.34; *q* = 0.031), the highest correlation values were found between male and female structures and they all correspond to positive correlations: epandrial posterior lobes with oviscapt pouches (λ = 6.09; *q* = 0.019; Fig. 6B), phallic spikes with oviscapt furrows (λ = 4.76; *q* = 0.017; Fig. 6C), phallic hook with vulval shield (λ = 3.10; *q* = 0.019; Fig. 6D), phallic spines with uterine shield (λ = 3.09; *q* = 0.019; Fig. 6D) and cercal teeth with ventral vaginal shields (λ = 3.11; *q* = 0.017; Fig. 6D). Interestingly, each of these coevolving structure pairs comes in contact with each other during copulation (see above). The male epandrial posterior lobes of the *melanogaster* and *yakuba* complexes did not show significant coevolution with the female oviscapt depressions which include both pouches and furrows, in these two complexes (λ = 2.01; *q* = 0.052), in concordance with the observation that the female pouches and furrows contact distinct male organs during copulation.

**Table 2:** Pagel’s (1994) phylogenetic correlations between male and female structures.

	Male							Female					
	EPL	LargeEPL	SmallEPL	Phallicspikes	Phallichook	Phallicspines	Cercalteeth	Oviscaptdepressions	Oviscaptpouches	Oviscaptfurrows	Vulvalshield	Uterineshield	Ventralshields
**Male**													
EPL	–	**0.046**	0.062	0.075	**0.019**	**0.031**	0.070	0.052	0.052	0.072	0.052	0.052	0.061
Large EPL	**2.25**	–	**0.031**	0.061	0.070	0.069	0.061	0.070	**0.019**	0.061	0.062	0.070	0.070
Small EPL	1.63	**3.34**	–	**0.019**	0.061	0.061	0.052	0.080	**0.019**	**0.019**	0.061	0.061	0.061
Phallic spikes	0.80	1.17	**2.84**	–	0.104	0.091	0.061	0.070	0.062	**<0.017**	0.100	0.080	**0.046**
Phallic hook	**2.00**	0.57	0.83	0.26	–	0.109	0.101	0.080	0.070	0.094	**0.019**	0.111	0.105
Phallic spines	**2.00**	0.57	0.83	0.26	0.13	–	0.095	0.070	0.070	0.111	0.108	**0.019**	0.091
Cercal teeth	0.90	0.61	1.23	1.75	0.13	0.13	–	0.080	0.061	**0.046**	0.080	0.083	**0.017**
**Female**													
Oviscapt depressions	2.01	0.79	0.44	1.39	0.94	0.93	0.65	–	**0.046**	0.070	0.070	0.062	0.075
Oviscapt pouches	0.97	**6.09**	**2.25**	1.24	0.57	0.57	0.63	**2.41**	–	0.062	0.070	0.061	0.061
Oviscapt furrows	0.80	1.17	**2.84**	**4.76**	0.27	0.27	**1.75**	1.40	1.17	–	0.100	0.083	0.061
Vulval shield	2.00	0.57	0.83	0.27	**3.10**	0.13	0.13	0.93	0.56	0.27	–	0.091	0.094
Uterine shield	2.00	0.57	0.83	0.27	0.13	**3.09**	0.13	0.93	0.57	0.27	0.18	–	0.091
Ventral shields	0.91	0.59	1.23	**1.75**	0.13	0.13	**3.11**	0.65	0.60	1.75	0.13	0.13	–

Likelihood differences (λ) are given below the diagonal, while FDR *q* values after 100 or 1000 simulations are given above the diagonal. Significant correlations (*q* ≤0.05) are given in bold; EPL: epandrial posterior lobes; oviscapt depressions: oviscapt pouches and oviscapt furrows.

## Discussion

### Species-specific Evolution of Female Genitalia

In contrast to previous reports [21], [22], our detailed investigation of female external genitalia in the *Drosophila melanogaster* species subgroup shows them to be both species-specific and coevolving with the male structures that they contact during copulation. We not only uncovered a correlation between male lobes and female pouches size (Fig. 2G), but also several qualitative associations between male and female genitalia: ventral vaginal shields and cercal teeth in *D. teissieri*, vulval shields and phallic hooks in *D. orena*, and uterine shields and large serrated phallus in *D. erecta* (Fig. 6D).

Our observations show that one cannot infer faster morphological evolution of genitalia in males than in females based on genitalia drawings in taxonomic literature, as descriptions of male structures are usually overrepresented in current literature [1], [2], [9]. Female genitalia of all species of the *melanogaster* subgroup except *D. yakuba* and *D. santomea* were previously drawn in taxonomic papers [32]–[36], [38], but only the oviscapt pouch of *D. simulans* [38] and the vulval shield of *D. orena* [35] were outlined. The *D. melanogaster* pouch can be seen on the SEM micrographs of Eberhard and Ramirez [22] and on the light micrographs of Kamimura [25] but the authors did not comment on it. The female genitalia traits that we uncovered here are either external depressions or internal sclerifications. These structures are not as conspicuous as the protrusions (epandrial posterior lobes, phallus spines, etc.) identified previously on male external and internal genitalia in the *D. melanogaster* subgroup species. Although *D. mauritiana* and *D. santomea* female genitalia did not display any species-specific sclerotized structures, their oviscapt exhibited other species-specific morphological traits, e.g. *D. mauritiana* oviscapts are larger, elongated and with stouter peg-like bristles (Fig. 1J, 3G, 6B, C).

Our observations also suggest that male- or female- specific structures located at similar anatomical positions might contact distinct female- or male-specific structures, respectively, in different species. For example, female pouches and furrows located at similar positions contact male lobes in the *melanogaster* species complex and phallic basal spikes in the *yakuba* species complex, respectively. Furthermore, the male phallic basal hooks contact a vaginal shield in *D. orena* whereas their corresponding structure in the *yakuba* complex, the basal spikes, contacts female furrows.

In our presently limited state of knowledge regarding the genetic and developmental basis of most of the genital traits described here, it is difficult to formulate homology hypotheses and to precisely determine whether similar traits have been lost or represent independent evolutionary innovations. For example, the various vaginal shields located at different positions in the female lower reproductive tract in diverse species may have diverged from a single ancestral shield or may be true independent innovations. We chose here to code each species-specific vaginal shield as an independent character, and the most parsimonious scenario associated with this view is thus multiple independent origins of the vaginal shield (Fig. 6D). Had we chosen to encode all vaginal shields as a single character state, then the most parsimonious scenario would have been a loss of vaginal shields in the ancestor of *D. yakuba* and *D. santomea*. Current data do not allow us to distinguish between these two possibilities. Similarly, oviscapt pouches might have originated independently in diverse species or might have been lost in *D. mauritiana* (Fig. 6B). Comparative work on the development of genitalia in the diverse *melanogaster* subgroup species is required to resolve this issue.

### Evolutionary Causes and Consequences of Male-female Genital Coevolution in *Drosophila*

At the post-copulatory level, intra- and interspecific size coevolution between male sperm and female sperm storage organs have been documented in *Drosophila* [55]–[57]. Given that several male seminal proteins are toxic to females [58], most notably the sex peptide which also controls sperm release from sperm storage organs [59], SAC has been proposed to be a major factor driving the rapid evolution of post-copulatory reproductive traits in *Drosophila*.

Our study reveals that female genital structures appear to coevolve with male structures in the *melanogaster* species subgroup. Such a pattern is consistent with the SAC hypothesis (antagonist coevolution), with the CFC hypothesis (cooperative evolution) and with another evolutionary hypothesis known as the lock-and-key [60], which posits that male and female genitalia coevolve rapidly to prevent or reduce copulation between closely-related species [61]. Divergence in genitalia morphologies is clearly not sufficient to prevent interspecific mating in the *melanogaster* species subgroup. Hybrids between *D. santomea* and *D. yakuba* have been found in natural populations on the island of São Tomé [62] and interspecific crosses can be performed in the laboratory between multiple species pairs in the *D. melanogaster* species subgroup [63].

In the lack of experimental data testing the costs induced to the female by the species-specific male characters identified here, it is difficult to conclude whether CFC or SAC is the prevalent force driving genital coevolution in the *melanogaster* subgroup. According to their anatomy and the male organs that they contact during copulation, the various vaginal shields discovered in this study might protect from apparently harmful phallic ornaments (in *D. erecta* and *D. orena*) or from cercal teeth (in *D. teissieri*) during copulation. These shields are devoid of grooves and furrows, suggesting that they might not facilitate genital coupling during copulation. Similarly, *D. yakuba* and *D. teissieri* oviscapt furrows might protect from harmful phallic spikes. Accordingly, contamination risk via matings wounds caused by these spikes in *D. yakuba* are higher in interspecific crosses with *D. santomea* females lacking oviscapt furrows than in intraspecific crosses [27]. The main force driving coevolution of lobes and pouches in the *melanogaster* complex is less clear. The oviscapt pouches may have evolved to screen males for the ones having the most compatible lobes or to help them grasp, in agreement with CFC. Alternatively, the oviscapt pouches and furrows may act as anti-grasping organs that help to dislodge the mating male. At present, it is difficult to interpret from comparative data alone the main driving force of lobe-pouch coevolution.

Recent experimental techniques such as laser surgery provide promising tools to understand the function and fitness consequences of microscopic genital structures. Experimental and genetic approaches have recently helped to understand the adaptive role of a few male grasping structures in *Drosophila* such as the mechanosensilla of the surstylus in *D. melanogaster* [64], the spine-like dorsal portion of the surstyli (known as secondary claspers) in *D. bipectinata* [65] and in *D. ananassae* [66], and the asymmetric epandrial lobes of *D. pachea* [67]. Alteration of these structures decreased male mating success, but the effect on female fitness was not determined. Future examination of the fitness consequences of experimental modifications of the male and female structures identified in this study would probably provide useful data to test which sexual selection hypothesis drives genitalia coevolution in the *melanogaster* species subgroup.

Theoretical models suggest that sexual selection on reproductive traits drives male and female coevolution along a line of equilibrium within populations, hence ultimately leading to populations differentiation and speciation [68]. However, empirical tests are lacking, probably due to the scarcity of cases where clearly coevolving male-female genital structures are known to vary in natural populations or between incompletely-isolated, nascent species. Geographical variation in male epandrial posterior lobes in the *melanogaster* complex [47] and in number of male cercal teeth in *D. teissieri* [43], [44] has been reported. Future analysis of the geographical variation of the corresponding coevolving female structures identified here might reveal interesting patterns.

With high-throughput sequencing methods and powerful genetic tools, the genes responsible for genitalia morphological differences between species of the *Drosophila melanogaster* subgroup are now within reach and should soon be identified. Having these data in hand will then allow us to explore important yet unanswered evolutionary questions, such as whether coevolving male and female traits share similar developmental basis and which selective forces drive male-female genitalia coevolution.

## Supporting Information

File S1 **A nexus file describing male and female genital characters distribution in the nine species of the** ***Drosophila melanogaster*** **species subgroup.**
(NEX)

## References

[1] Eberhard WG (1985) Sexual Selection and Animal Genitalia. Harvard University Press. 256 p.

[2] Eberhard WG (2010) Evolution of genitalia: theories, evidence, and new directions. Genetica 138: 5–18 doi:10.1007/s10709-009-9358-y. 19308664 10.1007/s10709-009-9358-y

[3] Stutt AD, Siva-Jothy MT (2001) Traumatic insemination and sexual conflict in the bed bug *Cimex lectularius* . Proc Natl Acad Sci USA 98: 5683–5687 doi:10.1073/pnas.101440698. 11331783 10.1073/pnas.101440698 PMC33273

[4] Arnqvist G, Rowe L (2005) Sexual Conflict: Princeton University Press. 360 p.

[5] Hosken DJ, Stockley P, Tregenza T, Wedell N (2009) Monogamy and the battle of the sexes. Annu Rev Entomol 54: 361–378 doi:10.1146/annurev.ento.54.110807.090608. 18793102 10.1146/annurev.ento.54.110807.090608

[6] Huber BA (1999) Sexual selection in pholcid spiders (Araneae, Pholcidae): artful chelicerae and forceful genitalia. J Arachnol 27: 135–141.

[7] Huber BA (2003) Southern African pholcid spiders: revision and cladistic analysis of *Quamtana* gen. nov. and *Spermophora* Hentz (Araneae: Pholcidae), with notes on male-female covariation. Zool J Linn Soc 139: 477–527.

[8] Huber BA (2005) High species diversity, male-female coevolution, and metaphyly in Southeast Asian pholcid spiders: the case of *Belisana* Thorell 1898 (Araneae, Pholcidae). Zoologica 155: 1–126.

[9] Eberhard WG (2004) Rapid divergent evolution of sexual morphology: comparative tests of antagonistic coevolution and traditional female choice. Evolution 58: 1947–1970. 15521454 10.1554/04-143

[10] Kulkarni CV (1940) On the systematic position, structural modifications, bionomics and development of a remarkable new family of cyprinodont fishes from the province of Bombay. Records of the Indian Museum: 379–423.

[11] Pratt HL (1979) Reproduction in the blue shark *Prionace glauca* . Fish Bull 77: 445–470.

[12] Rönn J, Katvala M, Arnqvist G (2007) Coevolution between harmful male genitalia and female resistance in seed beetles. Proc Natl Acad Sci USA 104: 10921–10925 doi:10.1073/pnas.0701170104. 17573531 10.1073/pnas.0701170104 PMC1904142

[13] Arnqvist G, Rowe L (2002) Antagonistic coevolution between the sexes in a group of insects. Nature 415: 787–789 doi:10.1038/415787a. 11845208 10.1038/415787a

[14] Carayon J (1966) Traumatic insemination and paragenital system. In: Usinger RL, editor. Monograph of Cimicidae (Hemiptera, Heteroptera). Entomol Soc America. 88–166.

[15] Brennan PLR, Prum RO, McCracken KG, Sorenson MD, Wilson RE, et al (2007) Coevolution of male and female genital morphology in waterfowl. PLoS ONE 2: e418 doi:10.1371/journal.pone.0000418. 17476339 10.1371/journal.pone.0000418 PMC1855079

[16] Evans JP, Gasparini C, Holwell GI, Ramnarine IW, Pitcher TE, et al (2011) Intraspecific evidence from guppies for correlated patterns of male and female genital trait diversification. Proc Biol Sci 278: 2611–2620 doi:10.1098/rspb.2010.2453. 21270040 10.1098/rspb.2010.2453 PMC3136825

[17] Simmons LW, Garcia-Gonzalez F (2011) Experimental coevolution of male and female genital morphology. Nat Commun 2: 374 doi:10.1038/ncomms1379. 21730955 10.1038/ncomms1379

[18] Vahed K (n.d.) Coercive copulation in the alpine bushcricket *Anonconotus alpinus* Yersin (Tettigoniidae: Tettigoniinae: Platycleidini). Ethology 108: 1065–1075.

[19] Peretti AV, Willemart RH (2006) Sexual coercion does not exclude luring behavior in the climbing camel-spider *Oltacola chacoensis* (Arachnida, Solifugae, Ammotrechidae). J Ethol 25: 29–39 doi:10.1007/s10164-006-0201-y.

[20] Hrušková-Martišová M, Pekár S, Bilde T (2010) Coercive copulation in two sexually cannibalistic camel-spider species (Arachnida: Solifugae). J Zool 282: 91–99 doi:10.1111/j.1469-7998.2010.00718.x.

[21] Jagadeeshan S, Singh RS (2006) A time-sequence functional analysis of mating behaviour and genital coupling in *Drosophila*: role of cryptic female choice and male sex-drive in the evolution of male genitalia. J Evol Biol 19: 1058–1070 doi:10.1111/j.1420-9101.2006.01099.x. 16780507 10.1111/j.1420-9101.2006.01099.x

[22] Eberhard W, Ramirez N (2004) Functional morphology of the male genitalia of four species of *Drosophila*: Failure to confirm both lock and key and male-female conflict. Annls Entomol Soc Am 97: 1007–1017.

[23] Dickson BJ (2008) Wired for sex: the neurobiology of *Drosophila* mating decisions. Science 322: 904–909 doi:10.1126/science.1159276. 18988843 10.1126/science.1159276

[24] Kamimura Y (2007) Twin intromittent organs of *Drosophila* for traumatic insemination. Biol Lett 3: 401–404 doi:10.1098/rsbl.2007.0192. 17519186 10.1098/rsbl.2007.0192 PMC2391172

[25] Kamimura Y (2010) Copulation anatomy of *Drosophila melanogaster* (Diptera: Drosophilidae): wound-making organs and their possible roles. Zoomorphology 129: 163–174 doi:10.1007/s00435-010-0109-5.

[26] Kamimura Y, Mitsumoto H (2012) Lock-and-key structural isolation between sibling *Drosophila* species. Entomol Sci 15: 197–201 doi:10.1111/j.1479-8298.2011.00490.x.

[27] Kamimura Y (2012) Correlated evolutionary changes in *Drosophila* female genitalia reduce the possible infection risk caused by male copulatory wounding. Behav Ecol Sociobiol 66: 1107–1114 doi:10.1007/s00265-012-1361-0.

[28] Lung O, Wolfner MF (1999) *Drosophila* seminal fluid proteins enter the circulatory system of the mated female fly by crossing the posterior vaginal wall. Insect Biochem Mol Biol 29: 1043–1052 doi:10.1016/S0965-1748(99)00078-8. 10612039 10.1016/s0965-1748(99)00078-8

[29] Ottiger M, Soller M, Stocker RF, Kubli E (2000) Binding sites of *Drosophila melanogaster* sex peptide pheromones. J Neurobiol 44: 57–71. 10880132

[30] Robertson HM (1988) Mating asymmetries and phylogeny in the *Drosophila melanogaster* species complex. Pacif. Sci. 42: 72–80.

[31] Kamimura Y, Mitsumoto H (2011) Comparative copulation anatomy of the *Drosophila melanogaster* species complex (Diptera: Drosophilidae). Entomol Sci 14: 399–410 doi:10.1111/j.1479-8298.2011.00467.x.

[32] Tsacas L (1971) *Drosophila teissieri*, nouvelle espèce africaine du groupe *melanogaster* et note sur deux autres espèces nouvelles pour l’Afrique (Dipt. Drosophilidae). Bull Soc Entomol Fr 76: 35–45.

[33] Tsacas L, Lachaise D (1974) Quatre nouvelles espèces de la Côte-d’Ivoire du genre *Drosophila*, groupe *melanogaster*, et discussion de l’origine du sous-groupe *melanogaster* (Diptera: Drosophilidae). Annls Univ Abidjan 7: 193–211.

[34] Tsacas L, David J (1974) *Drosophila mauritiana* n. sp. du groupe *melanogaster* de l’île Maurice. Bull Soc Ent Fr 79: 42–46.

[35] Tsacas L, David J (1978) Une septième espèce appartenant au sous-groupe *Drosophila melanogaster* Meigen: *Drosophila orena* spec. nov. du Cameroun. (Diptera: Drosophilidae). Beitr Ent 28: 179–181.

[36] Tsacas L, Bächli G (1981) *Drosophila sechellia*, n. sp., huitième espèce du sous-groupe *melanogaster* des Iles Sechelles (Diptera, Drosophilidae). Rev Fr Entomol 3: 146–150.

[37] Lachaise D, Harry M, Solignac M, Lemeunier F, Bénassi V, et al (2000) Evolutionary novelties in islands: *Drosophila santomea*, a new *melanogaster* sister species from São Tomé. Proc Biol Sci 267: 1487–1495 doi:10.1098/rspb.2000.1169. 11007323 10.1098/rspb.2000.1169 PMC1690712

[38] Bächli G, Vilela CR, Escher SA, Saura A, Bächli G, et al (2004) The Drosophilidae (Diptera) of Fennoscandia and Denmark. Fauna Entomol Scand 39: 1–362.

[39] Abramoff MD, Magalhães PJ, Ram SJ (2004) Image processing with ImageJ. Biophotonics Internat 11: 36–42.

[40] Pagel M (1994) Detecting correlated evolution on phylogenies: A general method for the comparative analysis of discrete characters. Proc Biol Sci 255: 37–45.

[41] Maddison WP, Maddison DR (2012) Mesquite: a modular system for evolutionary analysis. Version 2.75. Available:http://mesquiteproject.org.

[42] Obbard DJ, Maclennan J, Kim K-W, Rambaut A, O’Grady PM, et al (2012) Estimating divergence dates and substitution rates in the *Drosophila* phylogeny. Mol Biol Evol. 29: 3459–3473. 10.1093/molbev/mss150 PMC3472498 22683811

[43] Benjamini Y, Hochberg Y (1995) Controlling the false discovery rate: a practical approach to multiple testing. J R Stat Soc B 57: 289–300.

[44] Dalmasso C, Broët P, Moreau T (2005) A simple procedure for estimating the false discovery rate. Bioinformatics 21: 660–668. 15479710 10.1093/bioinformatics/bti063

[45] Coyne JA (1983) Genetic basis of differences in genital morphology among three sibling species of *Drosophila.* . Evolution 37: 1101–1118. 28556010 10.1111/j.1558-5646.1983.tb00225.x

[46] Coyne JA, Rux J, David JR (1991) Genetics of morphological differences and hybrid sterility between *Drosophila sechellia* and its relatives. Genet Res 57: 113–122. 2055454 10.1017/s0016672300029177

[47] Liu J, Mercer JM, Stam LF, Gibson GC, Zeng ZB, et al (1996) Genetic analysis of a morphological shape difference in the male genitalia of *Drosophila simulans* and *D. mauritiana* . Genetics 142: 1129–1145. 8846893 10.1093/genetics/142.4.1129 PMC1207113

[48] Macdonald SJ, Goldstein DB (1999) A quantitative genetic analysis of male sexual traits distinguishing the sibling species *Drosophila simulans* and *D. sechellia* . Genetics 153: 1683–1699. 10581276 10.1093/genetics/153.4.1683 PMC1460840

[49] Zeng Z-B, Liu J, Stam LF, Kao C-H, Mercer JM, et al (2000) Genetic architecture of a morphological shape difference between two *Drosophila* species. Genetics 154: 299–310. 10628989 10.1093/genetics/154.1.299 PMC1460924

[50] Masly JP, Dalton JE, Srivastava S, Chen L, Arbeitman MN (2011) The genetic basis of rapidly evolving male genital morphology in *Drosophila.* . Genetics 189: 357–374 doi:10.1534/genetics.111.130815. 21750260 10.1534/genetics.111.130815 PMC3176115

[51] Sánchez L, Santamaria P (1997) Reproductive isolation and morphogenetic evolution in *Drosophila* analyzed by breakage of ethological barriers. Genetics 147: 231–242. 9286683 10.1093/genetics/147.1.231 PMC1208107

[52] Lachaise D, Capy P, Cariou M-L, Joly D, Lemeunier F, et al. (2004) Nine relatives from one African ancestor: population biology and evolution of the *Drosophila melanogaster* subgroup species. In: Singh RS, Uyenoyama MK (eds.) The Evolution of Population Biology. Cambridge University Press. 315–343.

[53] Lachaise D, Lemeunier F, Veuille M (1981) Clinal variations in male genitalia in *Drosophila teissieri* Tsacas. Am Nat 117: 600–608.

[54] Joly D, Cariou M-L, Mhlanga-Mutangadura T, Lachaise D (2010) Male terminalia variation in the rainforest dwelling *Drosophila teissieri* contrasts with the sperm pattern and species stability. Genetica 138: 139–152 doi:10.1007/s10709-009-9423-6. 19921442 10.1007/s10709-009-9423-6

[55] Pitnick S, Markow T, Spicer GS (1999) Evolution of multiple kinds of female sperm-storage organs in *Drosophila.* . Evolution 53: 1804–1822 doi:10.2307/2640442. 28565462 10.1111/j.1558-5646.1999.tb04564.x

[56] Miller GT, Pitnick S (2002) Sperm-female coevolution in *Drosophila.* . Science 298: 1230–1233 doi:10.1126/science.1076968. 12424377 10.1126/science.1076968

[57] Joly D, Schiffer M (2010) Coevolution of male and female reproductive structures in *Drosophila.* . Genetica 138: 105–118 doi:10.1007/s10709-009-9392-9. 19657593 10.1007/s10709-009-9392-9

[58] Mueller JL, Page JL, Wolfner MF (2007) An ectopic expression screen reveals the protective and toxic effects of *Drosophila* seminal fluid proteins. Genetics 175: 777–783 doi:10.1534/genetics.106.065318. 17110486 10.1534/genetics.106.065318 PMC1800595

[59] Avila FW, Ravi Ram K, Bloch Qazi MC, Wolfner MF (2010) Sex peptide is required for the efficient release of stored sperm in mated *Drosophila* females. Genetics 186: 595–600 doi:10.1534/genetics.110.119735. 20679516 10.1534/genetics.110.119735 PMC2954482

[60] Dufour L (1844) Anatomie générale des Diptères. Annls Sci Nat 1: 224–264.

[61] Masly JP (2012) 170 years of “Lock-and-Key”: genital morphology and reproductive isolation. Internat J Evol Biol 2012: 1–10 doi:10.1155/2012/247352. 10.1155/2012/247352 PMC3235471 22263116

[62] Llopart A, Lachaise D, Coyne JA (2005) An anomalous hybrid zone in *Drosophila.* . Evolution 59: 2602–2607. 16526507

[63] Cariou ML, Silvain JF, Daubin V, Da Lage JL, Lachaise D (2001) Divergence between *Drosophila santomea* and allopatric or sympatric populations of *D. yakuba* using paralogous amylase genes and migration scenarios along the Cameroon volcanic line. Mol Ecol 10: 649–660. 11298976 10.1046/j.1365-294x.2001.01225.x

[64] Acebes A, Cobb M, Ferveur J-F (2003) Species-specific effects of single sensillum ablation on mating position in *Drosophila.* . J Exp Biol 206: 3095–3100 doi:10.1242/jeb.00522. 12878676 10.1242/jeb.00522

[65] Polak M, Rashed A (2010) Microscale laser surgery reveals adaptive function of male intromittent genitalia. Proc Biol Sci 277: 1371–1376 doi:10.1098/rspb.2009.1720. 20053645 10.1098/rspb.2009.1720 PMC2871932

[66] Grieshop K, Polak M (2012) The precopulatory function of male genital spines in *Drosophila ananassae* [Doleschall] (Diptera: Drosophilidae) revealed by laser surgery. Evolution 66: 2637–2645 doi:10.1111/j.1558-5646.2012.01638.x. 22834760 10.1111/j.1558-5646.2012.01638.x

[67] Lang M, Orgogozo V (2012) Distinct copulation positions in *Drosophila pachea* males with symmetric or asymmetric external genitalia. Contribs Zool 81: 87–94.

[68] Ritchie MG (2007) Sexual selection and speciation. Ann Rev Ecol Evol Syst 38: 79–102 doi:10.1146/annurev.ecolsys.38.091206.095733.

